# Sustainable Valorization of Peanut Byproducts: An Optimized Green Strategy for High-Yield Resveratrol Extraction

**DOI:** 10.1021/acsomega.6c02501

**Published:** 2026-06-29

**Authors:** Mirella T. F. Ranzeti, Gabriela Cremasco, Patrick J. Sherman, R. Dario Arrua, Cristiano S. Funari, Daniel Rinaldo

**Affiliations:** † Institute of Chemistry, São Paulo State University (UNESP), 14800-900 Araraquara, Brazil; ‡ Green Biotech Network, School of Sciences, São Paulo State University (UNESP), 17033-360 Bauru, Brazil; § Future Industries Institute, Mawson Lakes Campus, Adelaide University, Adelaide, South Australia 5095, Australia; ∥ Green Biotech Network, School of Agricultural Sciences, São Paulo State University (UNESP), Botucatu, 18610-034, Brazil; ⊥ Institute for Advanced Studies of Ocean (IEAMar), São Paulo State University (UNESP), Bauru 17033-360, Brazil

## Abstract

Peanut (*Arachis hypogaea* L.) roots represent a significant agroindustrial byproduct often discarded, despite being a potent source of resveratrol. This study proposes a sustainable strategy for the valorization of this biomass, aligning with the UN Sustainable Development Goal 12 (Responsible Consumption and Production). A “greener” extraction method was developed by combining Microwave-Assisted Extraction (MAE) with biobased Natural Deep Eutectic Solvents (NADES). Unlike conventional petrochemical solvents, the selected NADES (lactic acid and glycerol, 1:2) is derived from renewable sources and prepared through a 100% atom-efficient process. Optimization via Central Composite Design (CCD) yielded 563 ± 21 μg·g^–1^ of resveratrol (40 min, 60 °C), representing a 31.8-fold increase over conventional ethanolic maceration. The optimization process was critically assessed to strike a balance between extraction efficiency and environmental sustainability, ensuring high yields without excessive energy consumption. Greenness was quantified using AGREE and GAPI metrics, which demonstrated that the proposed method significantly reduces environmental impact compared to existing literature. These findings validate the use of peanut roots in a circular economy framework, offering a technically efficient and environmentally responsible alternative for the pharmaceutical and cosmetic industries.

## Graphical abstract

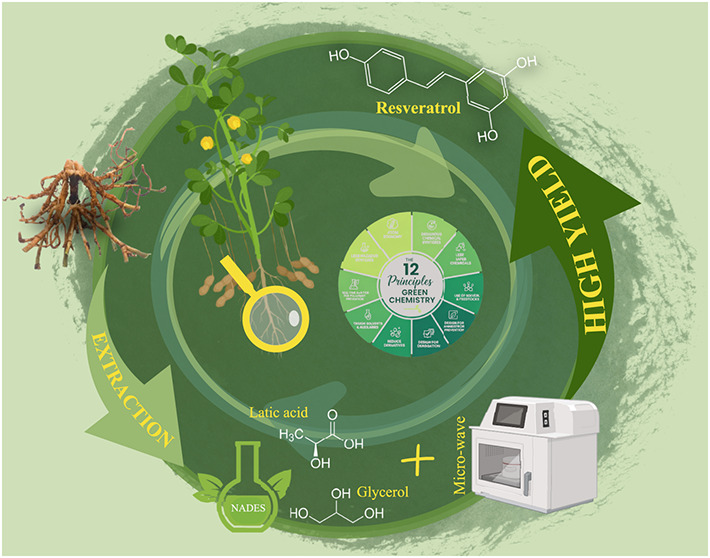

## Introduction

Peanuts (*Arachis hypogaea* L.), native to South America, are currently grown in virtually all tropical and temperate regions worldwide. They are one of the world’s leading oilseeds, ranking fourth in global production.^1^ In Brazil, peanuts are the sixth-largest agricultural crop, with production reaching 806,000 tons in 2024.^2^

Peanuts contain several flavonoids, phenolic acids, and flavonones of industrial interest, such as rutin, *p*-coumaric acid, caffeic acid, epicatechin, ferulic acid, quercetin, naringenin, and the stilbene resveratrol.^3,4^ Of particular interest is resveratrol, a highly bioactive metabolite with limited distribution in the plant kingdom but present in peanut roots, a byproduct of peanut seed production.^3^ Resveratrol exhibits antioxidant and anti-inflammatory activities and can also be used to protect the cardiovascular system. Additionally, this compound is a low-toxicity antitumor agent that can induce cell death in cancer cells, with anticancer potential.^3–5^

The exploitation of agricultural byproducts through green technologies is identified by the United Nations as an urgent need, aligning with several of its UN Sustainable Development Goals (SDGs).^6^ At the same time, an ecological exploration of these byproducts would help mitigate the problems associated with the accumulation of agricultural and forestry byproducts, adding value to production chains. In this sense, the initial obtaining of high-value-added metabolites from agricultural byproducts, which could be subsequently exploited for other purposes, presents itself as an alternative within a biorefinery and value-added chain approach.

A promising green approach is the development of selective and efficient extraction methods using natural deep eutectic solvents (NADES). Their most common combinations occur between choline chloride and glucose, citric acid, malic acid, propanoic acid, fructose, and others.^7^ NADESs can have certain advantages over common solvents, e.g., better extraction performance, biodegradability, and lower environmental impact and toxicity.^7,8^ They are also low-cost and can be tailored to the physical and chemical characteristics of the target compounds, making them a versatile and scalable solvent option.^8,9^ For example, for hydrophilic NADESs, a certain amount of water can be added to reduce their viscosity without destroying their supramolecular structure, thus facilitating mass transfer during the extraction.^9,10^

As a disadvantage, some NADES present high boiling points, thus making the separation from the extracted metabolites by drying difficult or requiring other strategies, such as separation by liquid–liquid extraction, solid-phase extraction using molecular sieves, or the use of hydrophobic resins or precipitation by the addition of antisolvents, such as water, or using an immunoaffinity column.^11^ Although the approaches mentioned lead to partial recovery of analytes from NADES, they all still have several constraints. However, NADES consisting of nontoxic compounds could be used to produce a “ready-to-use” extract.^11,12^

The green character of extraction methods using NADES as solvents can be enhanced by combining them with efficient extraction techniques, such as microwave-assisted extraction (MAE). MAE has two main mechanisms that ensure its high efficiency: rapid temperature increase, which reduces the medium’s viscosity and causes a large movement of ions.^13^

When combined with green techniques, the use of design of experiments (DoE) is a recommended approach for developing methods rationally and efficiently, with fewer experiments.^11^ Through mathematical models, it is possible to determine which variables are significant for a given process, as well as to evaluate the influences of those deemed significant through their responses, saving on experiments.^14^ In the extraction process development, response surface methodology (RSM) remains one of the most widely used optimization tools due to its ability to evaluate factor interactions and identify favorable operational conditions. More recently, advanced modeling approaches, including artificial neural networks (ANNs) and the group method of data handling (GMDH), have also been explored to improve predictive performance and process optimization in microwave-assisted extraction systems.^15^

The combination of green techniques and solvents with DoE enables compliance with GAC principles, aligning with the sustainable goals of the UN’s 2030 Agenda to achieve an efficient, low-cost, energy-efficient, and environmentally friendly extraction method.^6,16^

Given this context, this study aimed to develop a green, sustainable, and highly efficient method for extracting resveratrol from *A. hypogaea* (peanut) roots by using NADES as an extraction solvent. This study sought to simultaneously add economic value to this agroindustrial waste and minimize environmental impacts through the application of MAE associated with DoE for the rational optimization of experimental conditions.

## Results and Discussion

### Mapping Resveratrol in Peanut Roots

Initially, extraction methods from the literature for polar metabolites present in peanut roots (Chen^17^ and Lopes^18^) were reproduced to identify resveratrol by HPLC-DAD (Figures 1 and S1–S3) and LC-ESI-IT-MS^n^ (Figure S4). The UV spectrum and retention time (rt) were compared with those of the authentic standard by coinjection experiments.

**Figure 1:**
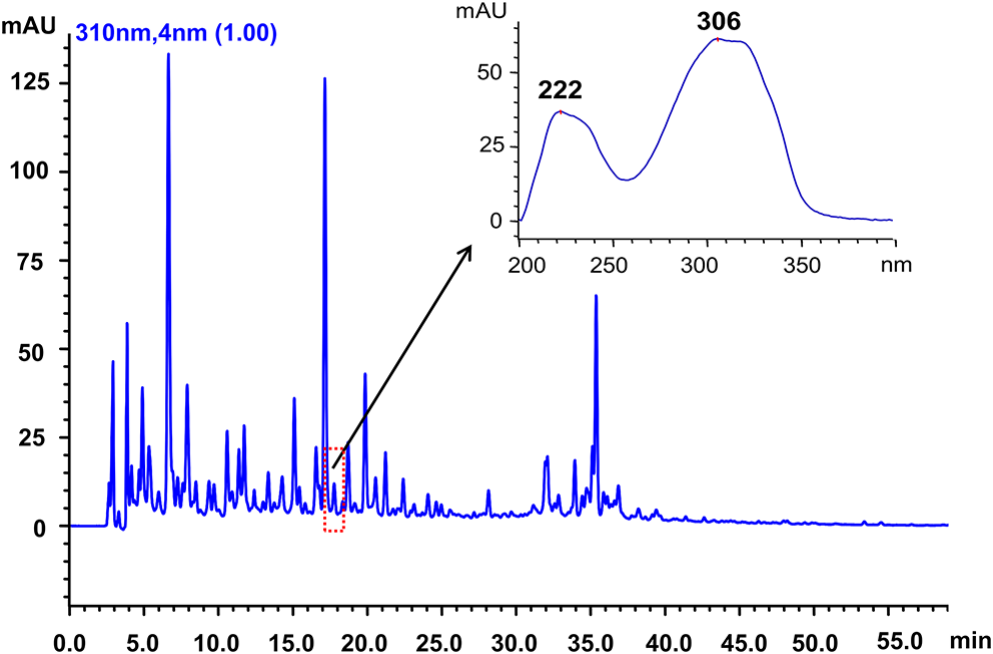
Representative HPLC-PDA/UV chromatograms of peanut byproducts (roots). Column: Phenomenex Luna (2) C18 column (250 mm × 4.6 mm × 5 μm); mobile phase: 0.1% TFA in H_2_O and ACN from 20 to 80% of ACN in 60 min; flow rate: 1.0 mL·min^–1^; analysis temperature: 30 °C; injection volume: 20 μL of nonconcentrated EtOH-H_2_O 8:2 (v/v) extract; monitored at 310 nm.

### Screening the Extractability of Different NADES

The extraction efficiency of the tested NADES was evaluated by the chromatographic peak area of resveratrol, where a larger area indicates superior performance. Blank analyses (pure NADES) were conducted under identical experimental conditions for background correction. NADES LT:GLY 1:1, mol/mol (N4), demonstrated the highest extraction yield (Figure 2 and Table S1). This superior performance is likely associated with physicochemical properties of the solvent system, including viscosity effects that can influence mass transfer during extraction and extract handling, as well as intermolecular interactions between the NADES components and the target analyte.^19^

**Figure 2:**
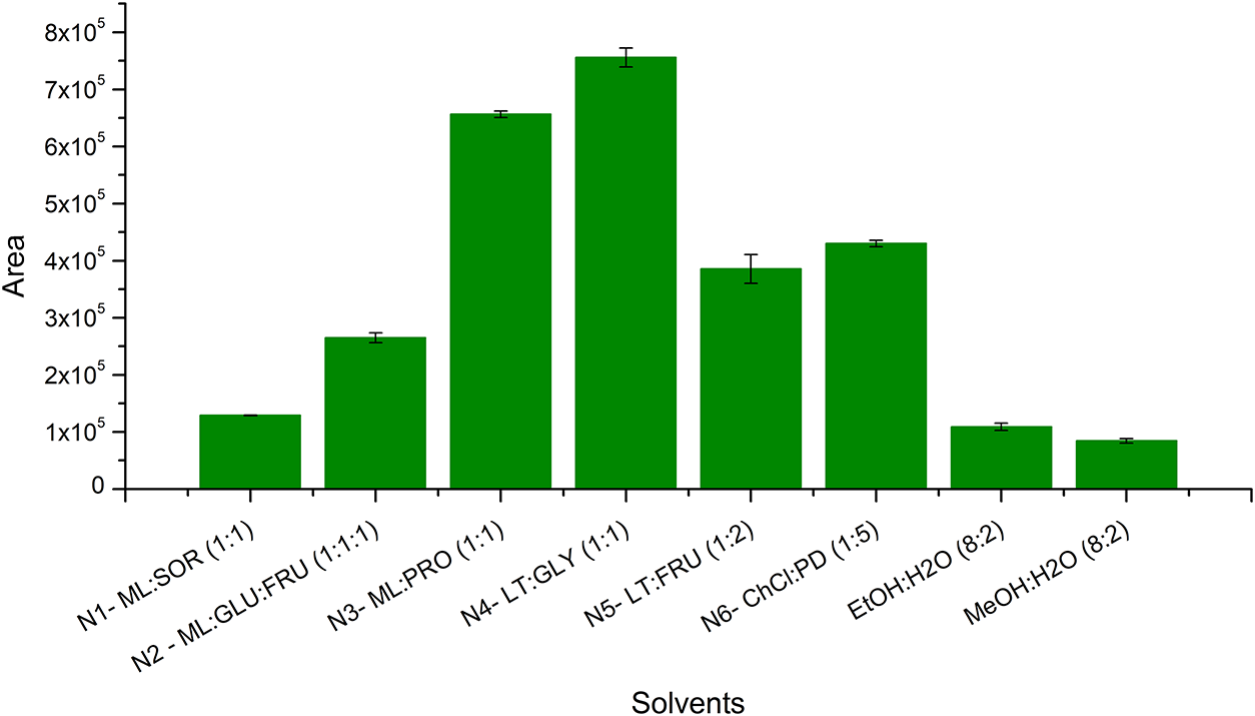
Comparison of the extractive performance of the tested NADES, expressed as the mean chromatographic peak areas of resveratrol (measured in mAU at 310 nm, *n* = 3).

Unlike conventional organic solvents (e.g., methanol), whose industrial synthesis depends on petrochemical precursors and high-enthalpy processes, the NADES used (LT:GLY) is prepared by a simple physical mixture of renewable components, with 100% atom economy and no byproduct generation, which minimizes the cradle-to-grave impact of the method.^20^

### Optimization of the NADES-MAE Extraction by DoE

The response recorded in the analyses of the 2^3^ full factorial design with five replicates at the central point was the chromatographic area of resveratrol (Table 1), evaluated at a significance level of 95%.

**Table 1:** Levels and Response Obtained in 2^3^ Full Factorial Design

	independent variables			response
experiments	*X*_1_ ^a^	*X*_2_ ^a^	*X*_3_ ^a^	total area^b^ (±%RSD)
1	–1 (0.02)	–1 (40 °C)	–1 (20 min)	952,527 ± 2.33
2	1 (0.08)	–1 (40 °C)	–1 (20 min)	675,361 ± 0.82
3	–1 (0.02)	1 (60 °C)	–1 (20 min)	1,067,823 ± 16.59
4	1 (0.08)	1 (60 °C)	–1 (20 min)	0 ± 0.00
5	–1 (0.02)	–1 (40 °C)	1 (60 min)	815,260 ± 21.33
6	1 (0.08)	–1 (40 °C)	1 (60 min)	729,733 ± 2.83
7	–1 (0.02)	1 (60 °C)	1 (60 min)	1,689,569 ± 11.25
8	1 (0.08)	1 (60 °C)	1 (60 min)	1,414,334 ± 24.55
9^c^	0 (0.05)	0 (50 °C)	0 (40 min)	986,596 ± 20.76
10^c^	0 (0.05)	0 (50 °C)	0 (40 min)	1,240,925 ± 3.58
11^c^	0 (0.05)	0 (50 °C)	0 (40 min)	1,132,607 ± 1.09
12^c^	0 (0.05)	0 (50 °C)	0 (40 min)	863,796 ± 8.79
13^c^	0 (0.05)	0 (50 °C)	0 (40 min)	964,307 ± 9.58

a *X*_1_, plant material/NADES ratio (m/v); *X*_2_, temperature (°C); and *X*_3_, extraction time (minutes).

b Values corresponding to 20 μL injection volume in the HPLC-DAD system.

c Central points.

The Pareto chart (Figure 3) demonstrated that the plant material/solvent ratio (*X*_1_) and extraction time (*X*_3_) had a statistically significant influence on the extraction yield. Conversely, temperature (*X*_2_) did not exhibit a significant effect (*p* > 0.05); however, the highest responses were empirically observed at the level +1 (60 °C, Table 2). Based on this empirical observation, the temperature was fixed at 60 °C for subsequent steps.

**Figure 3:**
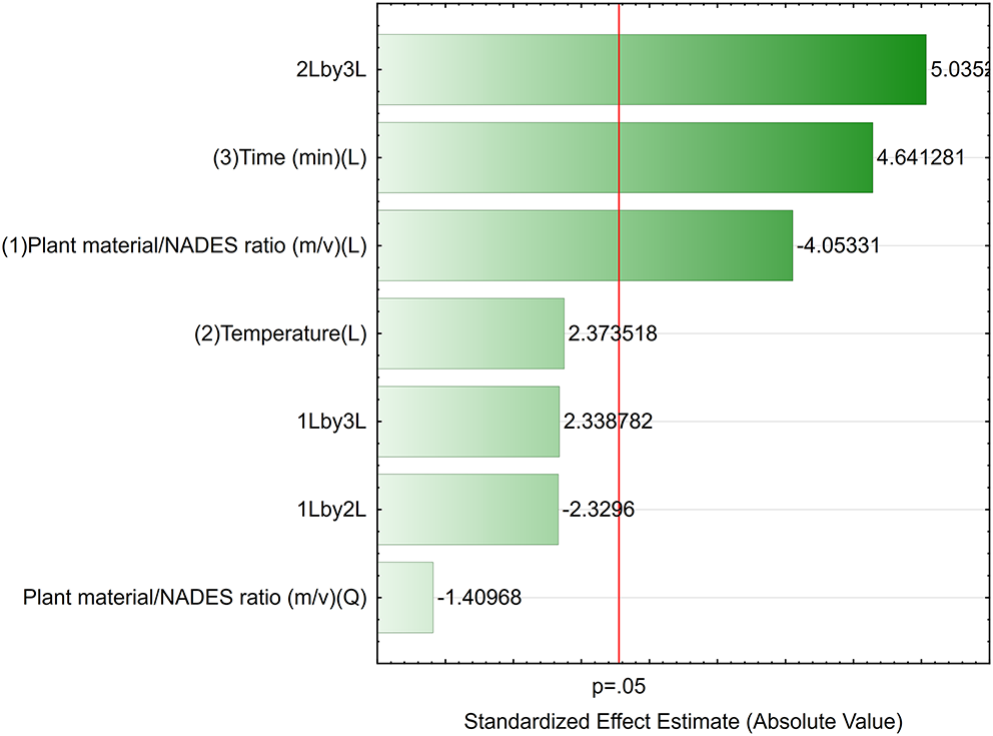
Pareto chart of FFD responses.

**Table 2:** Analysis of Variance (ANOVA) for the Estimated Model in the FFD

ANOVA				test *F*		
	quadratic sum	degrees of freedom	means square	calculated	tabulated	*p*-value
regression	1.81 × 10^12^	7	2.59 × 10^11^	9.77	4.88	0.0116
residue	1.33 × 10^11^	5	2.65 × 10^10^			
lack of fit	4.4 × 10^10^	1	4.4 × 10^10^	1.99	7.71	0.2314
pure error	8.85 × 10^10^	4	2.21 × 10^10^			
total	1.95 × 10^12^	12				
*R* ^2^	0.93					

curvature check						
	average	Ce^a^	variance	*s* ^b^	(+)	(−)
factorial	9.18 × 10^5^	–1.19 × 10^5^	7.2 × 10^9^	8.48 × 10^4^	–3.48 × 10^4^	–2.04 × 10^5^
CP^c^	1.04 × 10^6^					

a Ce: curvature estimative.

b *s*: standard deviation.

c CP: central point.

This decision was rationalized because 60 °C corresponds to the level associated with the highest empirical responses observed in the preceding step. Furthermore, fixing the temperature at this elevated level contributes to the reduction of the NADES viscosity and ensures greater resveratrol solubility. This approach allowed the optimization efforts to be directed exclusively toward the variables demonstrating the greatest influence of the plant material/NADES ratio (*X*_1_) and extraction time (*X*_3_). Consequently, the RSM model was built by considering only the significant variables, *X*_1_ and *X*_3_ (Figure 4).

**Figure 4:**
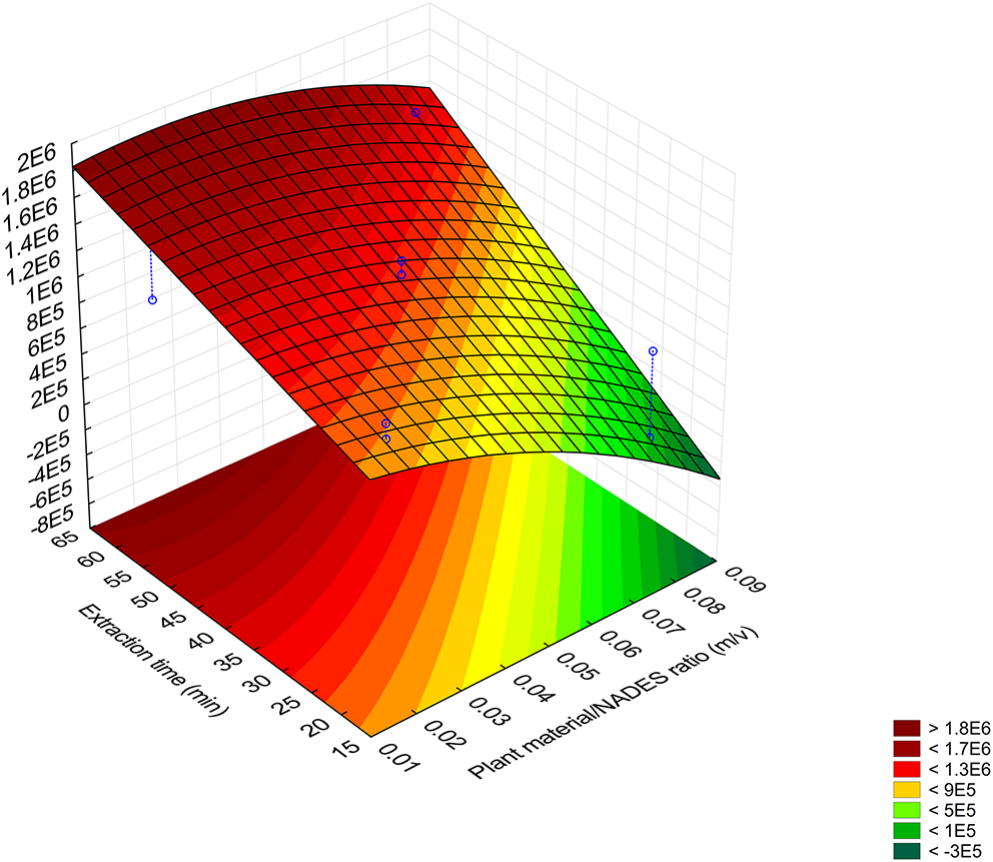
Response surface plot of the FFD with the variables plant material/NADES ratio (*X*_1_) and extraction time (*X*_3_).

Based on the geometry of the response surface (Figure 4) and the experimental data, a predictive mathematical model was derived from the optimization results, describing the relationship between the significant factors *X*_1_ and *X*_3_ (eq 1)­

1 $$A=1.2\times 10^{6}-6.6\times 10^{6}\;X_{1}+1.4\times 10^{4}\;X_{3}+2.1\times 10^{5}\;X_{1}\;X_{3}$$

where *A* is the total area of the peak (response variable), *X*_1_ represents the plant material/NADES ratio (w/v), and *X*_3_ is the extraction time (min).

The experimental data were evaluated using ANOVA to verify the model’s predictive capability and its adequacy in describing the obtained responses (Table 2). The highly significant regression term (*p* = 0.0116) confirmed that at least one factor exerts a relevant influence on the extraction yield. Furthermore, the nonsignificant lack of fit (*p* = 0.2314) validated the model’s consistency and its ability to accurately represent the relationship between the factors and the response variable. However, the statistically significant result of the curvature test indicates the presence of nonlinearity, justifying the necessary inclusion of quadratic terms in the final model to improve the overall adjustment and predictive power.

Thus, to optimize the experimental design, axial points were included for variables *X*_1_ (plant material/NADES ratio) and *X*_3_ (extraction time) according to the central composite design (CCD; Table 3). This approach allowed for a more comprehensive exploration of the response region of the significant variables and evaluation of potential quadratic effects, providing inputs for the construction of a more robust and predictive model.

**Table 3:** Chromatographic Area of the Resveratrol Band for CCD Experiments^a^,^b^,^c^

	independent variables		response
experiments	*X*_1_ ^a^	*X*_3_ ^a^	total area^b^ (±%RSD)
1	–1 (0.02)	–1 (20 min)	1,147,823 ± 6.45
2	1 (0.08)	–1 (20 min)	0 ± 0.00
3	–1 (0.02)	1 (60 min)	1,702,903 ± 10.41
4	1 (0.08)	1 (60 min)	1,081,000 ± 4.92
5	–α (0.0076)	0 (40 min)	248,584 ± 6.82
6	α (0.093)	0 (40 min)	783,197 ± 1.56
7	0 (0.05)	–α (11.7 min)	1,039,126 ± 2.06
8	0 (0.05)	α (68.3 min)	953,195 ± 11.13
9^c^	0 (0.05)	0 (40 min)	1,406,454 ± 7.06
10^c^	0 (0.05)	0 (40 min)	1,573,329 ± 3.70
11^c^	0 (0.05)	0 (40 min)	1,600,476 ± 1.27

a *X*_1_, plant material/NADES ratio (m/v); *X*_3_, extraction time (min).

b Values corresponding to 20 μL injection volume in the HPLC-DAD system.

c Central points.

The collected data were subsequently used to construct the response surface plot (Figure 5). This plot clearly illustrates a well-defined maximum response point within the experimental domain of investigation. This maximum visually represents the optimal operating conditions regarding *X*_1_ and *X*_3_, required to achieve the highest extraction efficiency.

**Figure 5:**
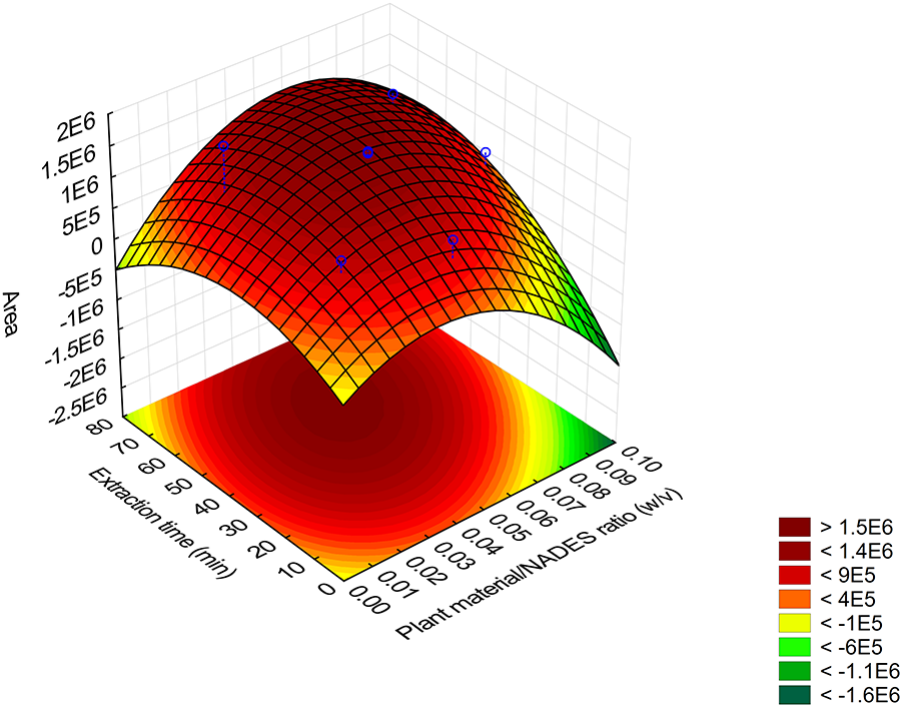
Response surface plot obtained by the CCD with the variables plant material/NADES ratio (*X*_1_) and extraction time (*X*_3_).

Based on these results, it was possible to calculate a new mathematical model, adjusted according to the central and axial points obtained in the CCD (eq 2)­

2 $$A=-2.75\times 10^{5}+3.61\times 10^{7}\;X_{1}-4.8\times 10^{8}\;{X_{1}}^{2}+4.03\times 10^{4}\;X_{3}-5.22\times 10^{2}\;{X_{3}}^{2}+2.19\times 10^{5}\;X_{1}\;X_{3}$$

This final model successfully incorporated the quadratic terms required to account for the observed curvature, which enables a more accurate and predictive description of the complex, nonlinear relationship between the significant variables (*X*_1_ and *X*_3_) and the extraction process response.

The full second-order model, obtained by the CCD, was then assessed using ANOVA at the 95% confidence level (Table 4). The results indicated, however, that the overall significance of the regression was not statistically achieved (*p* = 0.4009). Furthermore, the lack-of-fit test yielded a significant *p*-value (*p* = 0.0252 < 0.05), formally indicating that the quadratic model was not adequate to reliably predict the response throughout the investigated experimental domain. Therefore, the model should not be interpreted as a statistically validated predictive equation.

**Table 4:** Analysis of Variance (ANOVA) for the Model Estimated in CCD

ANOVA				test *F*		
	quadratic sum	degrees of freedom	means square	calculated	tabulated	*p*-value
regression	1.66 × 10^12^	5	3.31 × 10^11^	1.27	5.05	0.4009
residue	1.31 × 10^12^	5	2.62 × 10^11^			
lack of fit	1.29 × 10^12^	3	4.3 × 10^11^	38.85	19.16	0.0252
pure error	2.21 × 10^10^	2	1.10 × 10^10^			
total	2.97 × 10^12^	10				
*R* ^2^	0.56					

curvature check						
	average	Ce^a^	variance	*s* ^b^	(+)	(−)
factorial	8.69 × 10^5^	–6.57 × 10^5^	5.06 × 10^9^	7.11 × 10^4^	–5.86 × 10^5^	–7.28 × 10^5^
CP^c^	1.53 × 10^6^					

a Ce: curvature estimative.

b *s*: standard deviation.

c CP: central point.

Nevertheless, the experimental results revealed a consistent curvature tendency and a well-defined region of maximum response within the evaluated conditions, particularly associated with factors *X*_1_ and *X*_3_ (Figures S5 and 5). Thus, the response surface analysis was used only as a descriptive and exploratory tool to identify the experimental region associated with the highest extraction yield rather than to establish a predictive mathematical relationship.

Accordingly, the optimal MAE conditions reported here should be interpreted strictly as the best empirical conditions observed within the investigated domain: *X*_1_ = 0.05 (1:20 m/v), *X*_2_ = 60 °C, and *X*_3_ = 40 min.

### Analysis of Repeatability and Reproducibility of the Optimized NADES-MAE Method and Validation

The precision of the optimized NADES-MAE method was evaluated under the experimentally established optimum conditions by determining the % RSD of the resveratrol peak area. The method showed satisfactory precision, with the maximum % RSD obtained for repeatability (intraday precision) being 2.5% and for reproducibility (interday precision) being 3.8%.

Furthermore, the extraction performance at the experimentally established optimum condition was assessed by comparing experimental results (*n* = 9) with the value estimated by the RSM model (Figure S6). A % RSD of 2.98% was obtained, indicating good agreement between the experimental replicates and supporting the robustness of the extraction procedure under the selected conditions.

### Calibration Curve Validation and Comparative Performance against Conventional Extraction

The linearity of the developed method was successfully evaluated by constructing a calibration curve (*n* = 3, Figure 6). The relationship between peak area (*y*) and resveratrol concentration (*x*) was linear throughout the studied concentration range (eq 3). The resulting adjusted coefficient of determination (*R* ^2^) was 0.9995, which indicates that the linear model explains 99.95% of the total data variation, thereby confirming the excellent fit and linearity of the method

3 y=−24010.5+116289.9x

where *y* is the chromatographic area, and *x* is the concentration of resveratrol in the sample.

**Figure 6:**
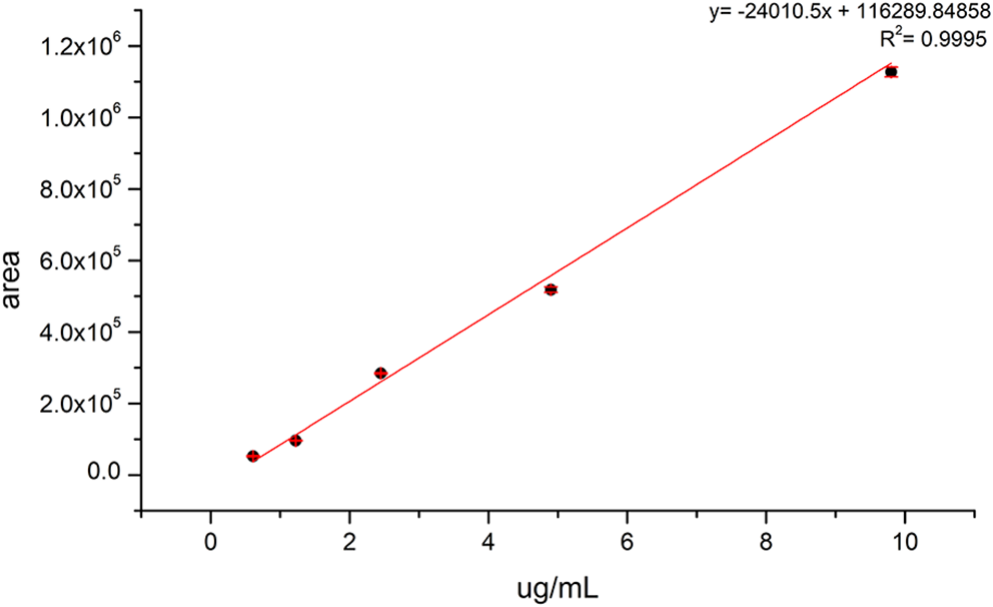
Calibration curve of resveratrol obtained by HPLC-DAD [chromatographic conditions described in “HPLC-DAD Analysis” Section].

Based on the validated calibration curve, the resveratrol concentration achieved in the extract using the developed and optimized NADES-MAE method was 0.563 ± 0.021 mg·g^–1^ of peanut root. The high reliability of this quantification is supported by the excellent *R* ^2^ value (0.9995) from the linearity assessment and the method’s parameter accuracy (Table S2). Furthermore, the determined LOQ ∼2.91 μg ensures the quantification is performed within the validated working range, lending high confidence to the reported result.

Crucially, the yield obtained by the developed method significantly surpassed those reported in the literature. Conventional methods described by Lopes^18^ and Chen, Wu, and Chiou^17^ achieved concentrations of 1.77 × 10^–2^ mg·g^–1^ and 9.32 × 10^–3^ mg·g^–1^, respectively. This demonstrates that the NADES-MAE approach achieved a resveratrol yield approximately 31.8 times higher than the best conventional method, confirming the successful and highly efficient recovery of resveratrol from the plant matrix using the sustainable NADES-based extraction.

It is important to emphasize that these results do not undermine the landmark findings of the reference methods cited in this work. Instead, our data suggest that the chemical potential of peanut roots can be further explored through contemporary green technologies. The observed differences likely stem from the superior extraction power of the MAE-NADES system, although it is acknowledged that experimental variations arising from our adaptation of the reference methods to local laboratory conditions, as well as natural biological differences in peanut cultivars, may also contribute to these discrepancies.

Despite the promising extraction performance of the MAE-NADES system, some limitations associated with the NADES should also be considered. The recovery of isolated analytes from these solvents remains challenging due to their low volatility, high viscosity, and strong hydrogen bonding network, which hinders solvent removal and analyte separation.^8^ However, since the present study aimed to obtain bioactive extracts rather than purified compounds, no purification step was performed. In this context, the low toxicity and biodegradable nature of NADES components may allow for the direct application of the obtained extracts without additional solvent removal steps.^21,22^

### Comparison of Efficiency and Green Credentials between the Developed Method and the Reference Methods

The efficiency of the optimized NADES-MAE method was rigorously compared with conventional methods reported in the literature (Figure 7 and Table S3). Although the method described by Lopes,^18^ which employed EtOH and water (8:2, v/v), demonstrated the highest efficiency among the conventional approaches, the NADES-MAE method achieved a superior yield. This result confirms the high extraction efficiency of the proposed approach for this specific application.

**Figure 7:**
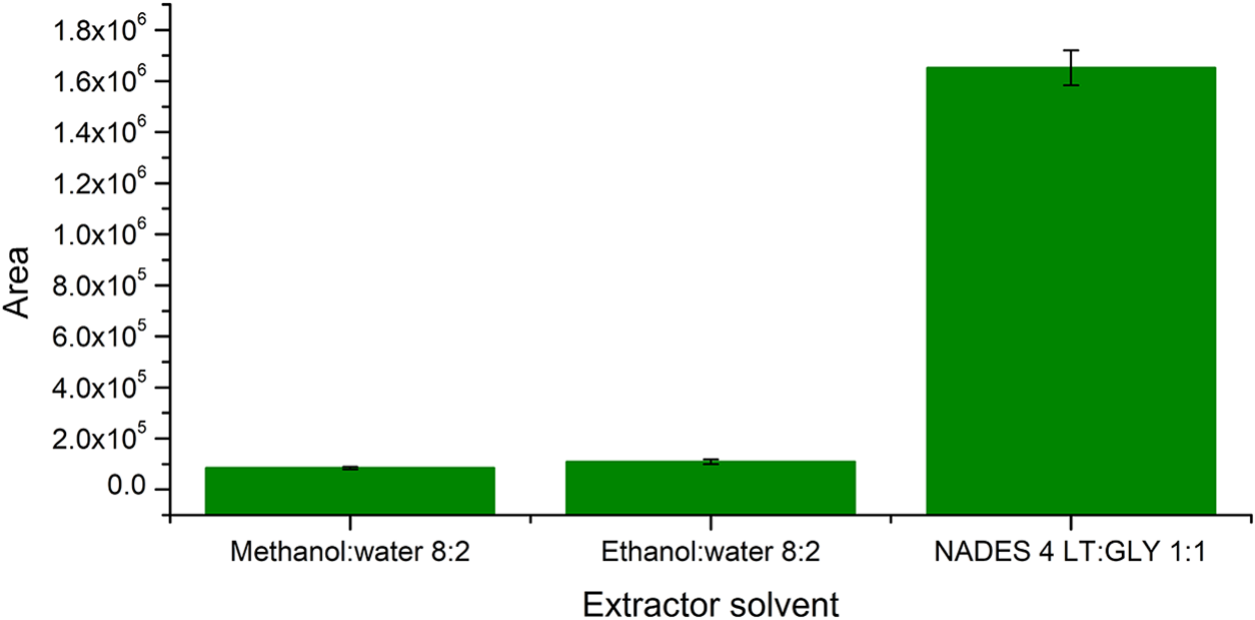
Comparison between extraction methods from the literature^17,18^ (*n* = 3) and extraction by NADES-MAE (*n* = 9).

While the compared methods employed different extraction techniques and solvent systems, the literature data were used as a reference to contextualize the extraction performance of the proposed MAE-NADES approach against that of established methodologies. Consequently, the green profile of the developed method was thoroughly evaluated by using consolidated analytical metrics, ensuring a comprehensive assessment of its environmental impact.

Assessment by the AGREE metric^23^ confirmed the superior green profile of the developed method, which yielded a score of 0.81 (Figure 8 and Table S4). This score, being closer to the maximum value of 1.0, indicates a high green character compared with the reference methods. The less favorable ratings (indicated by red and yellow sectors) were systemic limitations common to all three methods, stemming from constraints inherent in the final analytical technique. These penalties are specifically attributed to Parameter 1 (form of analysis), classified as nonideal due to requirement for offline analysis; Parameter 2 (sample quantity), penalized for the use of samples on a milligram scale (where nanoscale is considered ideal); and Parameter 3 *(in situ* performance), which received the lowest rating due to the necessary laboratory processing of the sample (*in vitro* performance).^23^

**Figure 8:**
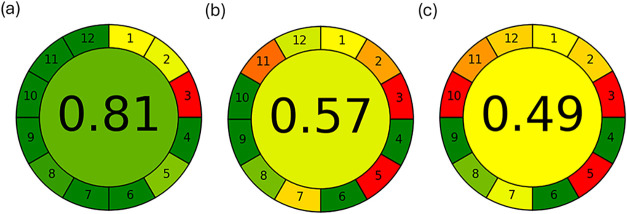
AGREE metric pictograms for (a) the developed method; (b) the LOPES^18^ method; and (c) the Chen^17^ method.

Furthermore, the GAPI Tool^24^ metric was applied to provide a detailed step-by-step assessment of the sustainability of the entire analytical procedure. The generated pictograms (Figure 9and Table S5) show that the NADES-MAE method presented a significantly higher number of green regions compared with the reference methods, thereby confirming its superiority in terms of environmental sustainability. The red color observed in the center of the pictogram is intrinsic to the sample treatment step, specifically, the mandatory requirement for an extraction process. It thus should not be interpreted as an indication of the unsustainability of the method. Similarly, the yellow area reflects the theoretical ideal of a complete absence of any extractive process, which is unachievable in this type of study.^24^

**Figure 9:**
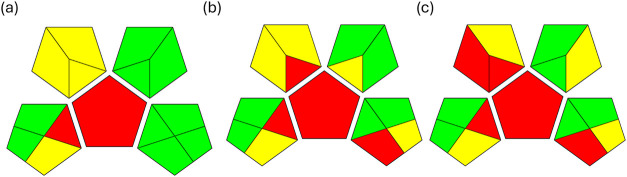
GAPI Tool metric for (a) the developed method; (b) the Lopes^18^ method; and (c) the Chen^17^ method.

The evaluation using AGREE and GAPI metrics confirmed that the superiority of the proposed method lies not only in the solvent but also in the integration of microwave-assisted extraction (MAE), which reduced the analysis time from hours to minutes compared to classical methods.^17,18^ This directly reflects the “energy efficiency” criterion of Green Chemistry, resulting in a score that surpasses methods based on Soxhlet or maceration.

## Conclusion

This study conclusively demonstrated that the lactic acid:glycerol (1:1, mol/mol) NADES, formulated with 20% water, represents a technically viable, highly efficient, and environmentally compatible alternative for extracting resveratrol from agroindustrial peanut root waste. The application of this mixture resulted in an efficiency significantly superior to conventional systems, such as MeOH:water (8:2, v/v)^17^ and EtOH:water (8:2, v/v).^18^

It is important to acknowledge that the green method developed in this study underwent a multivariate optimization process (DoE), which could, at first glance, suggest an unfair comparison with the univariate reference methods. However, the fundamental objective of this work is not simply to establish absolute quantitative superiority. Instead, it is to unequivocally demonstrate that the multivariate optimized method based on NADES-MAE can achieve a performance level comparable to, or—as demonstrated here—vastly superior to, traditional approaches while simultaneously offering a significantly more environmentally sustainable and technically viable alternative for high-value compound recovery.

## Experimental Section

### Reagents

The solvents ethanol (EtOH) HPLC grade, Merck, USA, and methanol (MeOH) HPLC grade, Merck, USA, were used for conventional extractions; acetonitrile (ACN) HPLC grade, Merck, USA, and trifluoroacetic acid (TFA) HPLC grade, Merck, USA, were used for chromatographic analysis. For coinjection with the standard, *trans*-resveratrol (Sigma-Aldrich) was used. 99% anhydrous caffeine standard (Sigma-Aldrich) was used as the internal standard in all chromatographic analyses. The following reagents, all from Sigma-Aldrich, were used to prepare the NADES: DL-malic acid (ML) ≥98%, lactic acid (LT) ≥85%, choline chloride (ChCL) ≥98%, d-sorbitol (SOR) ≥98%, D-(+)-glucose (GLU) ≥99.5%, GC, l-proline (PRO) ≥99%, HPLC, D-(−)-fructose (FRU) ≥99.5%, and glycerol (GLY) ≥99%, GC. In addition, 1.2-propanediol (PD) ≥99.5% from Vetec was used.

### Safety Information

The following solvents and reagents exhibit acute toxicity: ACN (Category 4), MeOH (Categories 1 and 3), EtOH (Category 2), and TFA (Category 4). In addition, ACN, MeOH, and EtOH solvents are considered to be flammable category 2. TFA is also corrosive (Category 1) and chronically toxic to aquatic environments (Category 3). The reagent GLY can cause severe eye irritation. Therefore, the solvents and reagents listed here require the use of PPE for handling in addition to caution. It is important to always have the SDS on hand in the case of accidents.

All information is sourced from safety data sheets provided by the Globally Harmonized System (GHS).

### Plant Material

Authentic roots of *A. hypogaea* were collected by Prof. Dr. Edvaldo Aparecido Amaral da Silva at the School of Agricultural Sciences, located in the city of Botucatu, SP, Brazil, corresponding to commercial cultivars from the State of São Paulo, referring to the 2022–2023 crop season.

### Sample Preparation

The residue was frozen in a freezer (Gelopar) at −20 °C for 48 h and then freeze-dried at −30 °C for 72 h in an α 1–2 LDplus freeze-dryer (Martin Christ). After freeze-drying, the material was pulverized in an industrial processor (Bermar). The root powder was sieved, and the fraction with particles between 250 and 850 μm was selected.^25^

### Reference Extractions

Conventional extraction methods described by Chen, Wu, and Chiou^17^ and Lopes^18^ were reproduced, with adaptations, using MeOH-H_2_O and EtOH-H_2_O, both at 8:2 (v/v) as reference solvents for extracting resveratrol from peanut roots.

In the reproduction of Chen, Wu, and Chiou’s^17^ method, 0.5 g of powdered plant material was subjected to dynamic maceration in 5 mL of MeOH/H_2_O (8:2, v/v) under constant stirring for 4 h at room temperature (25 °C). The mixture was then centrifuged in a 243 M FANEM centrifuge at 13,000 rpm, and the supernatant was filtered through a PTFE microfilter disc (0.45 μm, Phenomenex) for subsequent analysis by HPLC-DAD.

In the reproduction of Lopes’s^18^ method, the same experimental conditions described above were applied, replacing the extracting solvent with EtOH/H_2_O (8:2, v/v) and using 0.65 g of powdered plant material in 10 mL of solvent.

### HPLC-DAD Analysis

HPLC-DAD analyses were performed on a Shimadzu system (LC-20AT pump, DGU-20A5R degasser, SIL-20HT sampler, CTA-10AS VP column oven, and interface CBM-20A, Shimadzu, Kyoto, Japan), equipped with a Luna (2) C18, 250 mm × 4.6 mm, 5 μm column (Phenomenex, USA), and a photodiode array detector (DAD, SPD-M20A). The mobile phases were composed of water with 0.1% TFA (solvent A) and ACN (solvent B). The elution system was a gradient of 20–80% B in 60 min, with an injection volume of 20 μL, flow rate of 1 mL·min^–1^, and column temperature of 30 °C. The analyses were monitored at λ = 310 nm. The equilibration was achieved under the initial conditions of the gradient, with 20% B, a flow rate of 1 mL·min^–1^, and the column at 30 °C, for 15 min. The washout was 10 min, with 100% B passing through the column.

### Identification of the Target Compound

A standard solution of resveratrol at 1 mg·mL^–1^ in MeOH was prepared, and 20 μL of this was added to 1.5 mL of each extract. All samples containing the standard were analyzed via HPLC-DAD.

Standardized and nonstandardized extracts were analyzed by LC-ESI-IT-MS^n^, performed on Thermo Scientific, model LCQ Fleet, 4-channel Acella 600 pump, serial autosampler 751,214 high-performance liquid chromatography with electrospray ionization (ESI), source in negative mode, capillary voltage of 3.5 kV, drying gas flow of 10 L·h^–1^; nebulizer was 45 psig; the desolvation gas temperature was 280 °C, and the fragmentor voltage was 30 eV. N_2_ was used as the desolvation gas; helium was used as the collision gas. Version 2.0 of the XCalibur software (Thermo) was used for the acquisition and processing of experimental data.

### Preparation of Natural Deep Eutectic Solvents (NADES)

A total of 6 NADES (Table 5) were chosen according to their respective proportions and selectivities described in the literature, whose studies report their use for the extraction of medium- and high-polarity phenolic compounds. The NADES were prepared by dynamic maceration in the range of 50–80 °C, at 650 rpm, MR Hei-Tec (Heidolph), until homogeneous and transparent liquids were formed.^26^

**Table 5:** Investigated NADES for the Extraction of Resveratrol^a^

NADES	component 1	component 2	component 3	molar ratio	H_2_O (%)	coloration
N1	ML	SOR		1:1 ^27^	20	colorless
N2	ML	GLU	FRU	1:1:1 ^28^	20	slightly yellowish
N3	ML	PRO		1:1 ^9^	20	colorless
N4	LT	GLY		1:1 ^19^	20	colorless
N5	LT	FRU		1:2 ^26^	20	slightly yellowish
N6	ChCl	PD		1:5 ^29^	0	colorless

a ChCl = choline chloride; SOR = sorbitol; GLU = glucose; GLY = glycerol; ML = malic acid; PRO = proline; LT = lactic acid; FRU = fructose; and PD = 1,2-propanediol.

### Screening Step

Extractions were performed in triplicate by Microwave-Assisted Extraction (MAE) (Milestone Ethos Easy, DK). Powdered plant material and NADES were mixed at a 1:20 (w/w) ratio, irradiated for 1 h at 500 W power, under a controlled temperature of 50 °C.

After irradiation, the samples were centrifuged at 13,000 rpm (243 M FANEM, Brazil), and the resulting supernatants were filtered through a PTFE microfilter (0.45 μm, Phenomenex) and analyzed by HPLC-DAD.

### Design of Experiments

The 2^3^ full factorial design (FFD) was performed to identify statistically significant variables and evaluate model curvature. Subsequently, a central composite design (CCD) was employed to optimize the extraction only with the identified relevant variables in the FFD step.

The response variable utilized was the arithmetic mean of the peak area corresponding to resveratrol. The mean was obtained from three replicates (*n* = 3), with resveratrol identified in the HPLC-DAD chromatograms at λ = 310 nm.

The analysis of variance (ANOVA) calculations and response surface methodology (RSM) modeling were performed using Statistica software (Stat-Ease, Inc., Minneapolis, MN, USA). Complementary data and graphing were executed using Microsoft Excel 365 (Microsoft, Washington, DC, USA).

### Internal Standard

50.69 mg of caffeine was accurately weighed and dissolved in 50 mL of ethanol. Then, 150 μL of this stock solution was added to each 1.0 mL aliquot of both the extract and the resveratrol standard solution. This procedure resulted in a final, consistent caffeine concentration of 0.13 mg·mL^–1^ in all analyzed samples.

### Construction of the Calibration Curve and Limits of Detection/Quantification

For the quantification of resveratrol in the optimized extract, a calibration curve with five points was constructed by injecting the sample three times (6.13 × 10^–4^ mg·mL^–1^, 1.23 × 10^–3^ mg·mL^–1^, 2.45 × 10^–3^ mg·mL^–1^, 4.90 × 10^–3^ mg·mL^–1^, and 980 × 10^–3^ mg·mL^–1^ in EtOH). Additionally, a caffeine internal standard (1.32 × 10^–1^ mg·mL^–1^) was added to each point on the curve.

All solutions were analyzed by HPLC-DAD (*n* = 3) under the conditions described in the HPLC-DAD Analysis Section. The limit of detection (LOD) and limit of quantification (LOQ) were calculated according to the International Conference on Harmonization.^30^

### Repeatability and Reproducibility of the Optimized MAE-NADES Method

The precision of the method, encompassing both repeatability (intraday precision) and reproducibility (interday precision), was measured and evaluated according to ICH guidelines.^30^

Repeatability was assessed by performing and injecting three NADES-MAE extractions on the same day (intraday precision). To estimate interday precision, the optimized NADES-MAE extraction method was executed on three consecutive days, followed by the respective HPLC-DAD injections (conditions described in Section 1.5).

### Comparison of Efficiency and Environmental Impact with Conventional Methods in the Literature

The efficiency and environmental impact of the green method developed here were compared with two conventional methods from the literature, reproduced with adaptations (Reference Extractions Section).^17,18^

The environmental impact was assessed using the Analytical GREEnness Metric Approach and Software (AGREE)^23^ and Green Analytical Procedure Index (GAPI*)* ^24^ metrics, which are based on the 12 principles of GAC to assign comparative scores to the methods analyzed.

## Supplementary Material



## Notes

HPLC-DAD and LC-MS chromatograms, statistical data graphs, and tables with descriptions of the images and graphs (PDF)

## Abbreviations

ACN: acetonitrile
AGREE: analytical GREEnness metric approach and software
ANOVA: analysis of variance
CCD: central composite design
ChCL: choline chloride
DAD: diode array detector
DL: detection limit
DoE: design of experiments
ESI: electrospray ionization
EtOH: ethanol
FFD: full factorial design
FRU: fructose
GAC: green analytical chemistry
GAPI: green analytical procedure index
GLU: glucose
GLY: glycerol
HPLC: high-performance liquid chromatography
ICH: international conference on harmonization
λ: wavelength
LC: liquid chromatography
LT: lactic acid
MAE: microwave-assisted extraction
MeOH: methanol
ML: malic acid
NADES: natural deep eutectic solvents
NADES-MAE: NADES extracts made by MAE
PD: 1,2-propanediol
PRO: proline
PTFE: poly­(tetrafluoroethylene)
QL: quantification limit
RSM: response surface methodology
S: slope of the calibration curve
SDG: sustainable development goals
σ: standard deviation of the response
SOR: sorbitol
TFA: trifluoroacetic acid
tr: retention time
UN: United Nations
UV: ultraviolet
