# Supplementary material for: Sustainable Valorization of Peanut Byproducts: An Optimized Green Strategy for High-Yield Resveratrol Extraction
Source: ACS Omega. 2026 Jun 29;11(27):40195–204. doi: 10.1021/acsomega.6c02501 (PMC13382818; doi:10.1021/acsomega.6c02501)
Supplement: Supplementary file 1 [file ao6c02501_si_001.pdf]

SUPPLEMENTARY MATERIAL

SUSTAINABLE VALORIZATION OF PEANUT BY-PRODUCTS: AN OPTIMIZED GREEN STRATEGY FOR HIGH-YIELD RESVERATROL EXTRACTION

Mirella Thaisa Ferreira Ranzeti,[a,b] Gabriela Cremasco,[b] Patrick James Sherman,[c] Ruben Dario Arrua,[c] Cristiano Soleo de Funari,[d] Daniel Rinaldo [a,b,e]

[a]Institute of Chemistry, São Paulo State University (UNESP), 14800-900 Araraquara, Brazil

[b]Green Biotech Network, School of Sciences, São Paulo State University (UNESP), 17033-360 Bauru, Brazil

[c]Future Industries Institute, Mawson Lakes Campus, University of South Australia, Adelaide, SA 5095, Australia

[d]Green Biotech Network, School of Agricultural Sciences, São Paulo State University (UNESP), Botucatu, 18610-034, Brazil

[e]Institute for Advanced Studies of Ocean (IEAMAR), São Paulo State University (UNESP), Bauru, 17033-360, Brazil

Corresponding Author: Daniel Rinaldo; Phone +55 14 3103-9814; E-mail daniel.rinaldo@unesp.br

Figures S1-S3 show the chromatograms of the *trans*-resveratrol standard analyses and the methanolic and ethanolic extracts of peanuts, as well as the co-injection of the extract with the standard. Figures S4 and S5 show the Pareto chart and the desirability chart obtained by central composite design, respectively.

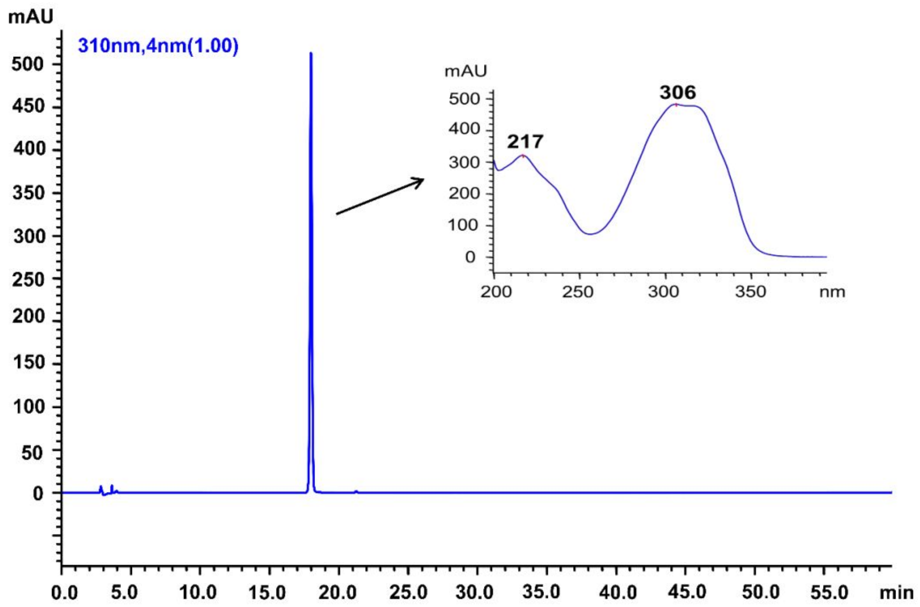

Figure S1. Chromatogram of the *trans*-resveratrol standard and its respective UV spectrum [analyzed by HPLC-DAD; 0.1% TFA (mobile phase A) and ACN (mobile phase B) 20-80% gradient; time of 60 min; injection volume of 20 µL; flow rate of 1 mL.min^{-1}; temperature of 30 °C; λ = 310 nm].

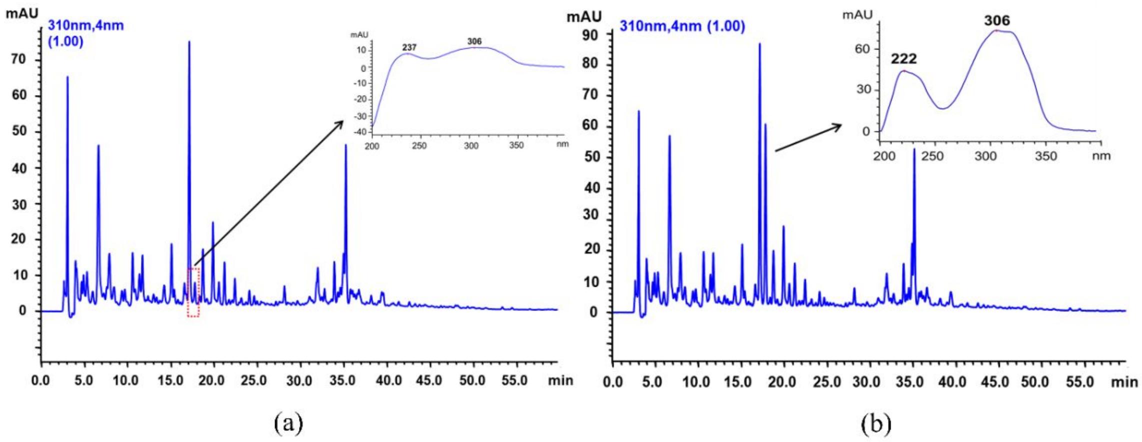

Figure S2. Chromatogram of extracts from the Chen *et al*. [17] method (a) pure and its respective UV spectrum of resveratrol and (b) with trans-resveratrol standard and its respective UV spectrum [analyzed by HPLC-DAD; 0.1% TFA (mobile phase A) and ACN (mobile phase B) 20-80% gradient; time of 60 min; injection volume of 20 µL; flow rate of 1 mL.min^{-1}; temperature of 30 °C; λ = 310 nm].

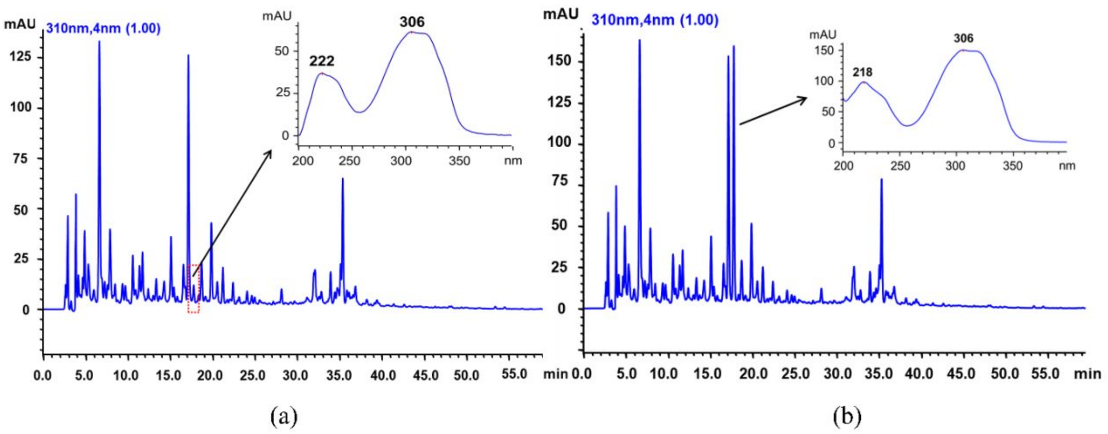

Figure S3. Chromatogram of extracts from the Lopes method [18] (a) pure and its respective UV spectrum of resveratrol and (b) with trans-resveratrol standard and its respective UV spectrum [analyzed by HPLC-DAD; 0.1% TFA (mobile phase A) and ACN (mobile phase B) 20-80% gradient; time of 60 min; injection volume of 20 μL; flow rate of 1 mL.min^{-1}; temperature of 30 °C; λ = 310 nm].

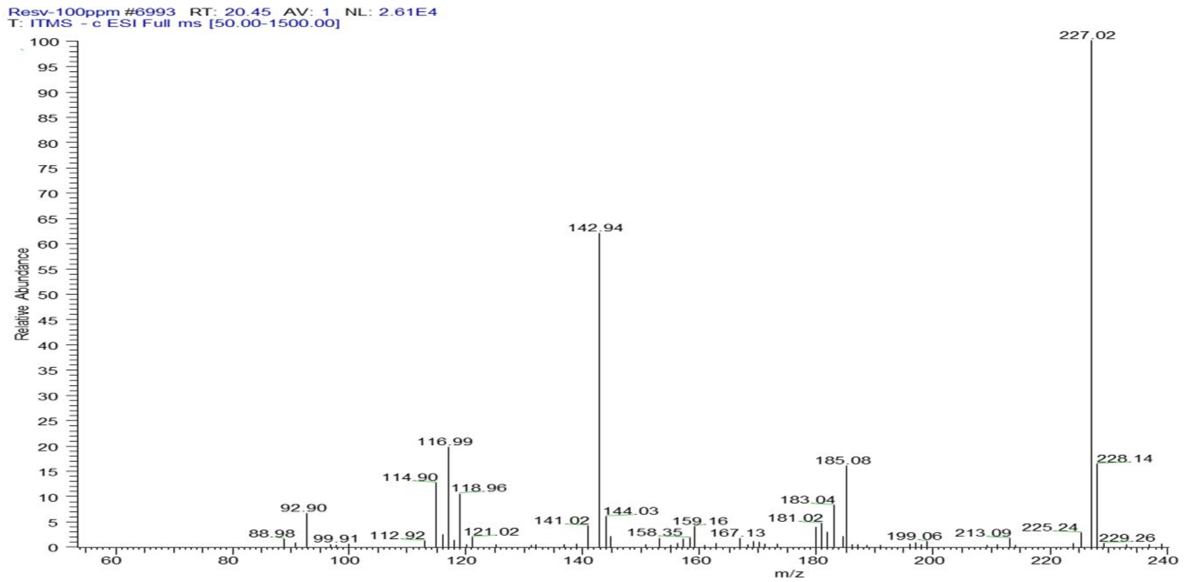

Figure S4. Mass spectrum of resveratrol (*m/z*) present in peanut extract [analyzed by LC-ESI-IT-MS^n; negative mode; capillary voltage of 3.5 kV; drying gas flow of 10 $L.h^{-1}$; 45 psig; desolvation gas (N_2) temperature of 280 ^oC; fragmentor voltage of 30 eV; helium as the collision gas].

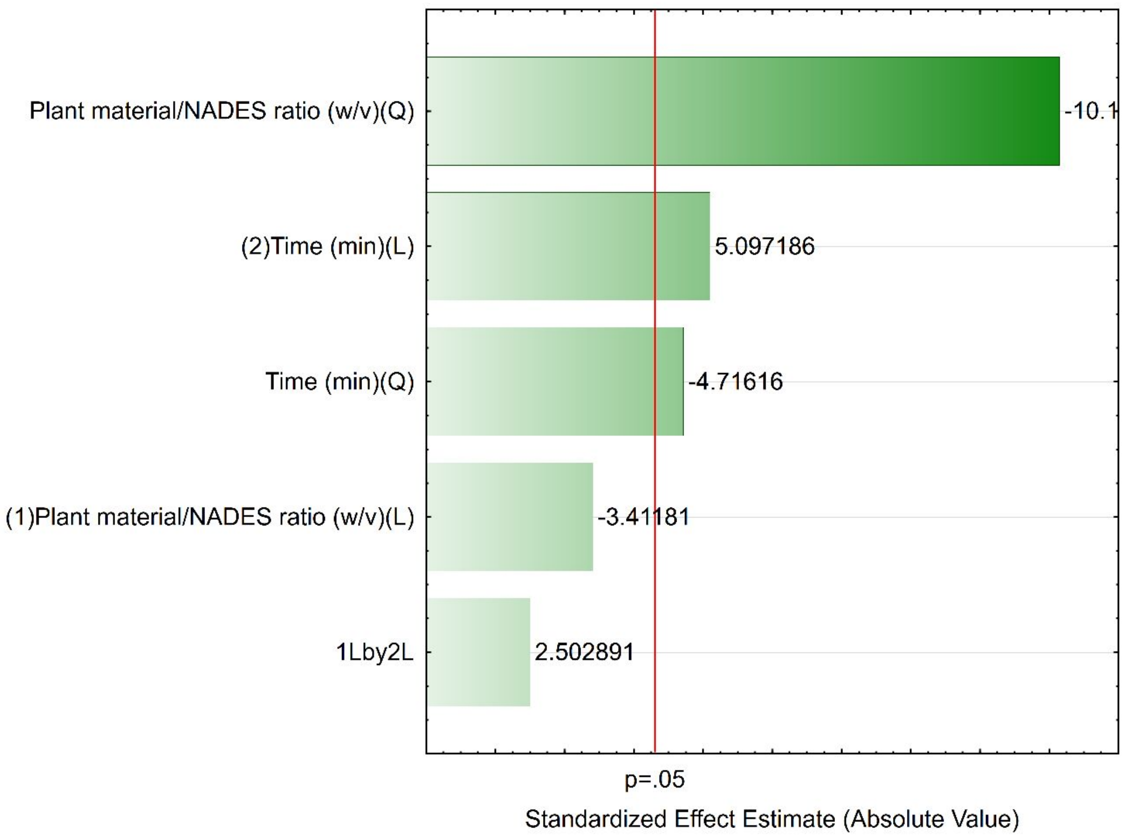

Figure S5. Pareto chart obtained from the responses of the central composite design.

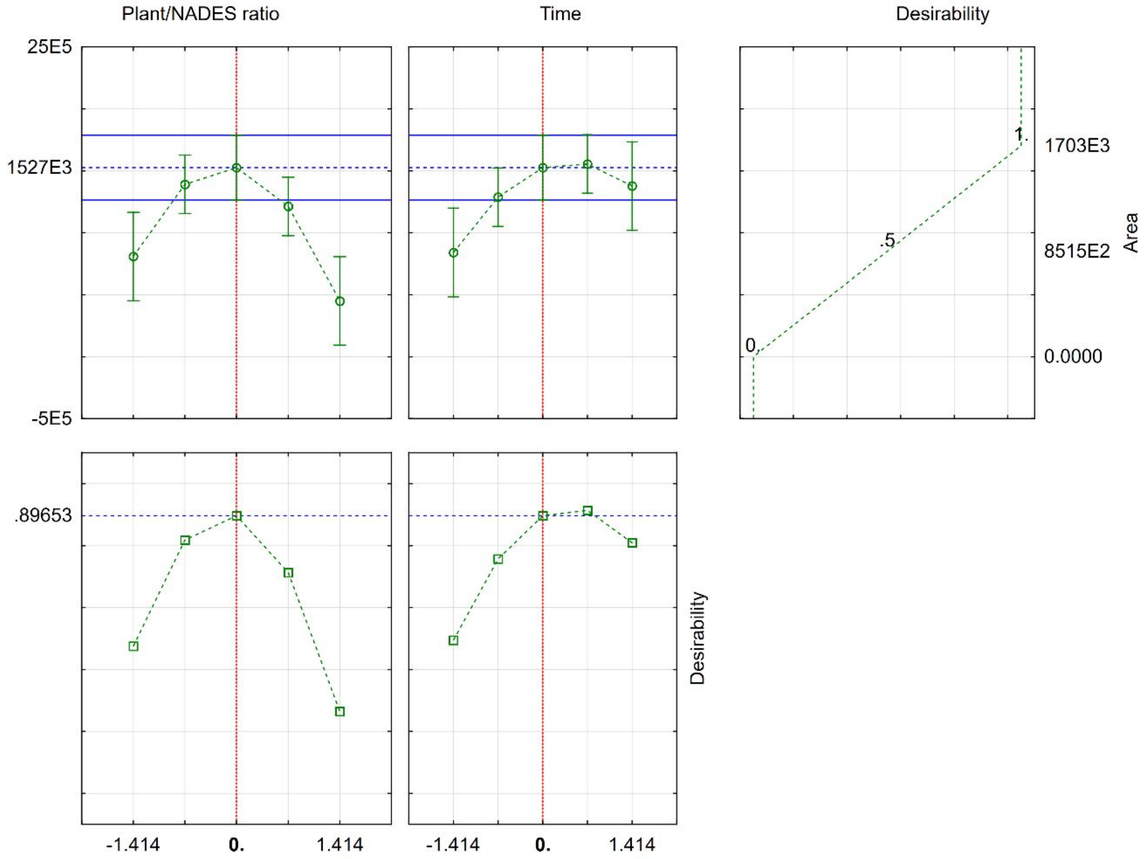

Figure S6. Desirability chart for the optimal levels of variables X_1 and X_3.

Below are the resveratrol areas from the NADES screening (Table S1), data from the calibration curve graph for the quantification of resveratrol in the samples (Table S2), the comparison between the methods in the literature and the method developed (Table S3), and the respective scores in the AGREE (Table S4) and GAPI Tool metrics (Table S5).

Table S1. Average of resveratrol chromatographic peak areas obtained by HPLC-DAD for extractions from peanut [n=3]

NADES	**Total area** (±%RSD)
N1 – ML:SOR 1:1	129326 ±0.45
N2– ML:GLU:FRU 1:1:1	264956 ±3.18
N3 – ML:PRO 1:1	656411 ±0.89
N4 – LT:GLY 1:1	755950 ±2.20
N5 – LT:FRU 1:2	385488 ±6.53
N6 – ChCl:PD 1:5	430035 ±1.29

ChCl=choline chloride; SOR=sorbitol; GLU=glucose; GLY=glycerol; ML=malic acid; PRO=proline; LT=latic acid; FRU=frutose; PD=1,2-propanediol.

Table S2. Parameters obtained from the calibration curve equation for resveratrol quantification (Figure 6).

Terms	Values	Error
a	-24010.50	33855.77
b	116289.85	6693.21
QL	0.96 $\mu g.mL^{-1}$	
DL	2.91 $\mu g.mL^{-1}$	

a = slope of the line; b = intercept of the line; LOQ = limit of quantification; LOD = limit of detection.

Table S3. Comparison of the extraction methods reported in the literature [17,18] with extraction by MAE-NADES.

Extractor solvent	Technique used	Total area (%RSD)
Methanol:water 8:1	Dynamic maceration	84420 ±5.9
Ethanol:water 8:1	Dynamic maceration	109091 ±2.8
NADES LT:GLY 1:1	MAE	1762257 ±4.1

GLY=glycerol; LT=lactic acid.

Table S4. Input parameters in the AGREE metric software to elucidate the pictograms referring to Figure 7.

GAC Principles	(a)	(b)	(c)
1- sample treatment	0.48	0.48	0.48
2- sample size	0.59	0.32	0.41
3- in situ measurements	0.00	0.00	0.00
4- analytical process	1.00	1.00	1.00
5- automated and miniaturized methods	0.75	0.25	0.25
6- derivatization	1.00	1.00	1.00
7- analytics management	1.00	0.39	0.48
8- multianalyte methods	0.73	0.73	0.73
9- use of energy	1.00	0.91	0.91
10- used reagents	1.00	1.0	0.00
11- toxic reagents	1.00	0.20	0.27
12- safety of the operator	1.00	0.60	0.40

Table S5. Input parameters in the GAPI metric software to elucidate the image, referring to Figure 8.

Parameters	(a)	(b)	(c)
Collection	Off-line	Off-line	Off-line
Preservation	Chemical or physical	Chemical or physical	Chemical or physical
Transport	None	None	None
Storage	Under normal conditions	None	None
Type of method	Extraction	Extraction	Extraction
Scale	Micro	Macro	Macro
Solvents	Green	Green	Non-green
Additional	Simple	Simple	Simple
Amount	<10 mL (<10 g)	10-100 mL (10-100 g)	<10
Health	NFPA=0 or 1		
Safety	NFPA= 0 or 1		
Energy	<=0.1 Kw per sample		
Occupational	Hermetic sealing		
Waste	>1 mL (> 1g)		
Waste treatment	Recycling		
